# The impact of preanalytical variables on the analysis of cell-free DNA from blood and urine samples

**DOI:** 10.3389/fcell.2024.1385041

**Published:** 2024-05-09

**Authors:** Hongwei Peng, Ming Pan, Zongning Zhou, Congbo Chen, Xing Xing, Shaoping Cheng, Shanshan Zhang, Hang Zheng, Kaiyu Qian

**Affiliations:** ^1^ Department of Biological Repositories, Human Genetic Resources Preservation Center of Hubei Province, Hubei Key Laboratory of Urological Diseases, Zhongnan Hospital of Wuhan University, Wuhan, China; ^2^ Taihe Skills Training Center, Taihe Hospital, Hubei University of Medicine, Shiyan, Hubei, China; ^3^ Department of Urology, Taihe Hospital, Hubei University of Medicine, Shiyan, Hubei, China; ^4^ Department of Urology, The First Affiliated Hospital of Yangtze University, Jingzhou, Hubei, China; ^5^ Department of Urology, Laboratory of Precision Medicine, Zhongnan Hospital of Wuhan University, Wuhan, China

**Keywords:** cell-free DNA (cfDNA), preanalytical variables, standardization, blood, urine

## Abstract

Cell-free DNA (cfDNA), a burgeoning class of molecular biomarkers, has been extensively studied across a variety of biomedical fields. As a key component of liquid biopsy, cfDNA testing is gaining prominence in disease detection and management due to the convenience of sample collection and the abundant wealth of genetic information it provides. However, the broader clinical application of cfDNA is currently impeded by a lack of standardization in the preanalytical procedures for cfDNA analysis. A number of fundamental challenges, including the selection of appropriate preanalytical procedures, prevention of short cfDNA fragment loss, and the validation of various cfDNA measurement methods, remain unaddressed. These existing hurdles lead to difficulties in comparing results and ensuring repeatability, thereby undermining the reliability of cfDNA analysis in clinical settings. This review discusses the crucial preanalytical factors that influence cfDNA analysis outcomes, including sample collection, transportation, temporary storage, processing, extraction, quality control, and long-term storage. The review provides clarification on achievable consensus and offers an analysis of the current issues with the goal of standardizing preanalytical procedures for cfDNA analysis.

## 1 Introduction

Cell-free DNA (cfDNA) was first discovered in the peripheral blood of healthy individuals in 1948 (Mandel and Metais, 1948). Subsequent research demonstrated elevated levels of cfDNA in patients with cancer (Song et al., 2022; Wu et al., 2022) and inflammation (Schneck et al., 2017), indicating that cfDNA analysis could be a valuable tool for health monitoring (Hayashi et al., 2019). Originating from apoptotic or necrotic events and active release mechanisms in the presence of intracellular circulating nucleases (Qi et al., 2023), cfDNA, with its modal size around 166 base pairs (bp) in plasma, has been linked to nucleosomal structures (Lo et al., 2021). Due to the stability of cfDNA molecules in body fluids (Polini et al., 2019), their wealth of genetic and epigenetic information (Wu et al., 2019; Xiao et al., 2022), and the noninvasive or minimally invasive nature of body fluid collection for cfDNA analysis (Ellervik and Vaught, 2015), cfDNA is considered an ideal biomarker for disease prevention, diagnosis, treatment, and prognosis (Szilagyi et al., 2020). Fetal noninvasive prenatal testing (NIPT) based on cfDNA levels was the first successful application of cfDNA in health monitoring (Schmid et al., 2018). Recent studies have explored the role of cfDNA in various areas, including NIPT (Zhu et al., 2021), cancer (Nabet et al., 2020), diabetes (Humardani et al., 2023), cardiovascular diseases (Polina et al., 2020), organ transplantation (Wolf-Doty et al., 2021), autoimmune diseases (Mondelo-Macia et al., 2021), and sepsis (Lenz et al., 2022). However, the progress of most related studies remains in the preliminary stage, likely due to the challenges presented by preanalytical variables.

**FIGURE 1:**
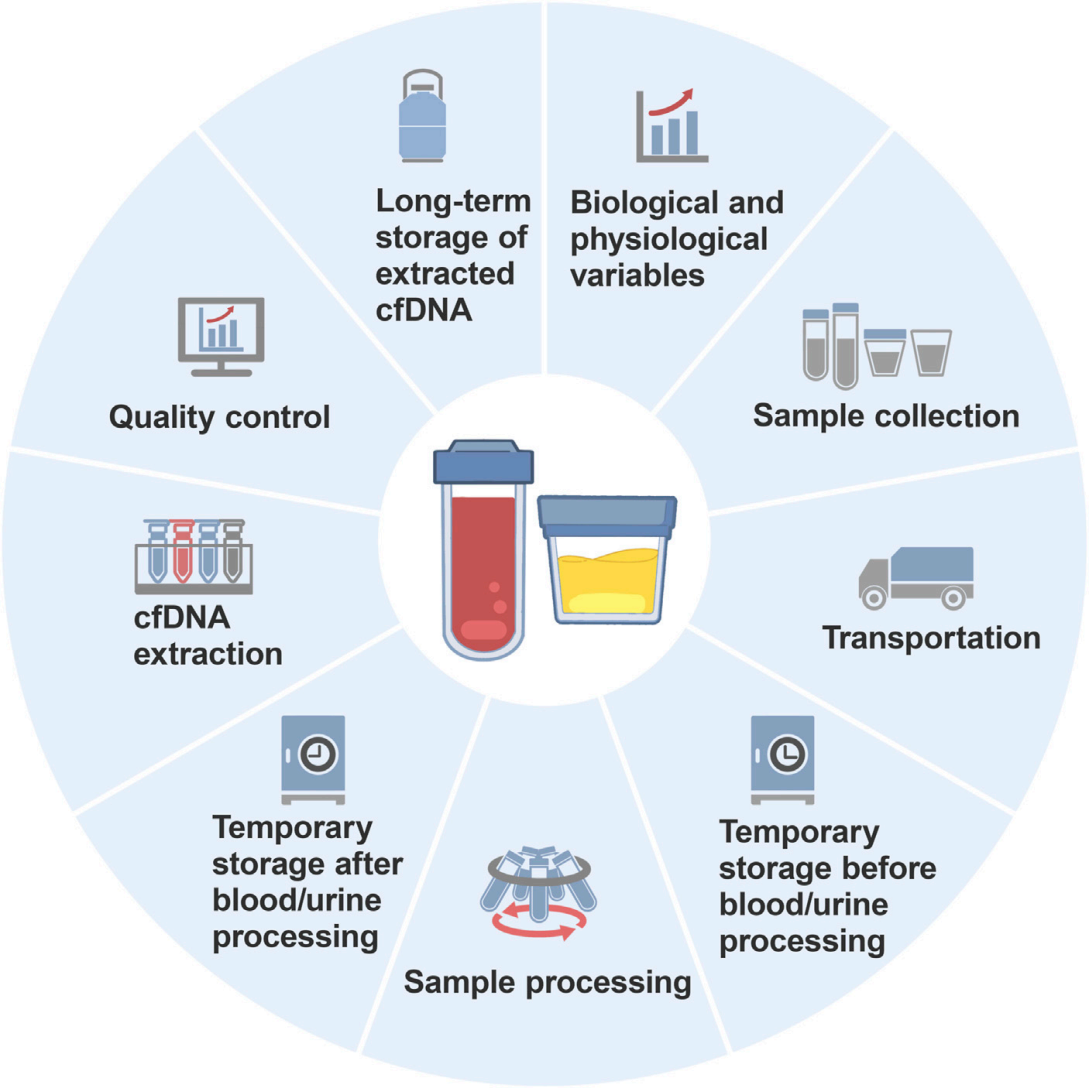
Spectrum of preanalytical procedures affecting the analysis of cell-free DNA.

The journey from sample collection to cfDNA analysis is intricate and involves several steps, such as preparation, collection, transportation, temporary storage, processing, extraction, quality control, and long-term storage (Figure 1). Each step involves numerous conditions or details, and the variables interact with each other. Moreover, many studies inadequately describe the preanalytical variables for cfDNA analysis in their Materials and Methods sections (Campbell et al., 2015; Wolf-Doty et al., 2021; Shen et al., 2022), leading to questionable credibility of analytical results and inefficiency in method verification. Diao et al. surveyed the quality assurance (the questionnaire included preanalysis, postanalysis and performance validation for mNGS) of metagenomic next-generation sequencing (mNGS) used for detecting microbial cfDNA in blood samples across 80 laboratories in China and found significant variation in the mNGS workflow among the laboratories (Diao et al., 2022). Specifically, the sequencing platforms used in the mNGS laboratories included 49 Illumina laboratories, 16 Beijing Genomics Institute laboratories, 13 Ion Torrent laboratories and 2 Nanopore sequencing laboratories, and the interpretation standards for the mNGS results were inconsistent among the laboratories. Consequently, establishing widely applicable standards and consensuses presents a formidable challenge.

Blood and urine samples are valuable resources in biomedical research. Over the past few years, progress has been made in some effective methods (e.g., EDTA tubes, specialized collection tubes and specialized kits) (Salvianti et al., 2020; Ungerer et al., 2020; Deger et al., 2021; Wever et al., 2022) and in establishing preliminary guidelines for preanalytical variables (Meddeb et al., 2019). However, with the advancement of technology and new insights into unresolved issues, the existing consensus on preanalytical variables for blood-derived cfDNA analysis needs to be updated. cfDNA in urine has shown great potential in noninvasive diagnosis. Urine is an ideal biomaterial for the study of urological diseases due to its direct contact with the urinary system and convenient collection of sufficient volume (Ruppert et al., 2023). Research has shown significantly higher levels of urine cfDNA (ucfDNA) in patients with urinary tumors compared to healthy individuals (Nikanjam et al., 2022). Nevertheless, the clinical application of ucfDNA is relatively rare, possibly due to its sensitivity to environmental conditions (e.g., temperature and pH level of preservation solution) (Kim et al., 2023; Ruppert et al., 2023), which makes it easier to degrade rapidly, resulting in inadequate concentrations for downstream analysis (Nel et al., 2023) compared to blood-derived cfDNA. Therefore, the weaker stability makes ucfDNA analysis more susceptible to complex preanalytical variables, highlighting the importance of establishing a consensus on ucfDNA experimental procedures.

Clear documentation of the key details and preanalytical variables in experimental procedures is important, as it forms the basis for discussion and analytical results. Such detailed information is of paramount importance for consensus building. In this review, we delve into the preanalytical variables affecting cfDNA analysis. We clarify the achievable consensus in preanalytical variables and analyze existing challenges with the aim of standardizing preanalytical procedures for cfDNA analysis.

## 2 Preanalytical variables affecting cfDNA analysis

### 2.1 Biological and physiological variables prior to sample collection

The characteristics of cfDNA in biospecimens are influenced by many biological and physiological variables before collection. These variables are often interrelated and subject to significant intra- and inter-individual differences (Ungerer et al., 2020). However, few of these variables have been individually studied, resulting in a limited understanding of their specific impacts on cfDNA characteristics. Potential biological and physiological variables (Table 1) that may affect cfDNA characteristics mainly include demographic differences (e.g., age and gender) (Aucamp et al., 2018; Lin et al., 2021), living habits (e.g., diet and exercise) (Aw et al., 2018; Huminska-Lisowska et al., 2021), psychophysical state (e.g., obesity, stress and emotion) (Trumpff et al., 2019; Drag and Kilpelainen, 2021; Ampo et al., 2022), origin (Stejskal et al., 2023), physiological process (e.g., menstruation and pregnancy) (Bianchi and Chiu, 2018; Yuwono et al., 2022), infection (Arshad et al., 2018), pathological diseases (e.g., diabetes, cancer, dysimmunity, and inflammation) (Fatouros et al., 2010; Bronkhorst et al., 2019; Cheng et al., 2020; Humardani et al., 2023), therapy (Muller Bark et al., 2020) and surgery (Oellerich et al., 2021). To shed light on how these variables influence cfDNA characteristics, representative examples, such as cfDNA origin mechanisms and cfDNA in cancer, are discussed below.

**TABLE 1:** Biological and physiological variables affecting cfDNA analysis.

Variables	Explanations	References
Gender	The yields of cfDNA in women are higher than that in men	Lin et al. (2021)
Age	Compared with young people, the cfDNA levels in the elderly (over 60 years old) are significantly higher	Aucamp et al. (2018)
Diet	Changes in dietary composition may determine the types and amount of mitochondrial cfDNA	Aw et al. (2018)
Exercise	Changes of cfDNA are associated with tissue injury induced by exercises	Huminska-Lisowska et al. (2021)
Obesity	Obesity results in higher cfDNA concentration by inducing inflammation	Drag and Kilpelainen (2021)
Stress	Acute psychological stress may affect mitochondria and cause an increase in circulating cell-free mtDNA	Trumpff et al. (2019)
Emotion	The levels of cf-mtDNA in plasma of elderly patients with depression and frailty is increased	Ampo et al. (2022)
Origin	cfDNA derived from apoptosis is finally digested into fragments of 166 bp or integer multiples of 166 bp. Necrosis usually leads to the existence of cfDNA fragments larger than 10,000 bp. cfDNA derived from living cells has a wide range of fragment sizes including 1,000–3,000 bp and 30–20,000 bp	Ungerer et al. (2020)
Pregnancy	The cfDNA levels are increased as gestation progresses and peak before labor	Bianchi and Chiu (2018)
Infection	People living with HIV have higher cf-mtDNA levels than their uninfected peers	Arshad et al. (2018)
Diabetes	Dietary exposure triggers apoptosis-induced proliferation in adipocytes for diabetic patients, and lead to cfDNA release	Humardani et al. (2023)
Cancer	The increased cfDNA level usually depends on the increased tumor size and growth rate in early cancer	Bronkhorst et al. (2019)
Dysimmunity	Abnormal immune status are associated with carcinogenesis which was detected and analysed in ctDNA	Cheng et al. (2020)
Inflammation	Exercise-induced inflammation increases the levels of cfDNA, and the amount of cfDNA depends on the severity of inflammation	Fatouros et al. (2010)
Therapy	As a biomarker of many diseases, the content of cfDNA changes with the therapy of diseases	Muller Bark et al. (2020)
Surgery	In renal transplant patients, the level of donor-derived cfDNA increased due to graft injury	Oellerich et al. (2021)

cfDNA originates from various sources, and its characteristics vary greatly, often identifiable based on DNA fragment length. Currently, several major mechanisms of cfDNA origin are recognized, including passive release from apoptotic (Handayani et al., 2023) and necrotic cells (Jahr et al., 2001) and active release from living cells (Thakur et al., 2014). During apoptosis, nucleosomes, composed of histone octamers and double-stranded DNA wrapped around the protein complex, are released into the blood and sheared by various nucleases to form cfDNA (Duplessis et al., 2018; Fedyuk et al., 2023). Consequently, cfDNA fragments resulting from apoptosis are typically 160–180 bp or 360 bp in length, consistent with the length of the nucleosome (Jahr et al., 2001; Markus et al., 2022). In contrast, cfDNA fragments from necrotic cells are usually larger than 10,000 bp (Fujihara et al., 2021). Furthermore, living cells can actively secrete cfDNA in various forms of extracellular vesicles, containing cfDNA fragments ranging from 150 to 6,000 bp (Thakur et al., 2014; Fernando et al., 2017) and even up to two million bp (Vagner et al., 2018).

Despite different origin mechanisms, the cfDNA fragment ranges in blood and urine are largely regular due to metabolic equilibrium. Plasma cfDNA is predominantly split into 166 bp fragments, as confirmed by precise sequencing technologies (Jiang et al., 2015; Hudecova et al., 2022). ucfDNA, filtered through the renal barrier or directly released into urine following apoptosis and necrosis of urogenital cells (Cimmino et al., 2021), displays a wider range of lengths (Jain et al., 2019). Large molecular fragments, usually more than 1,000 bp, mainly originate from the necrosis of exfoliated uroepithelial cells or leukocytes (Streleckiene et al., 2018). Conversely, small molecular fragments, usually 10–150 bp and 150–200 bp (Melkonyan et al., 2008), mainly originate from apoptotic cells in the bloodstream.

Circulating tumor DNA (ctDNA), a subtype of cfDNA, is released by necrotic or apoptotic tumor cells and carries a wealth of genetic information related to tumorigenesis and progression (Weng et al., 2020). Increased ctDNA levels typically correlate with increased tumor size and growth rate in early-stage cancer (Fiala and Diamandis, 2018). Generally, cancer patients exhibit higher plasma ctDNA levels than healthy individuals (Xu et al., 2021). Apart from presenting significant intraindividual and interindividual variation, ctDNA levels in malignant tumors are significantly greater than those in nonmalignant tumors (Thierry et al., 2016). In summary, ctDNA levels vary greatly across different cancer stages and can be efficiently used to detect alterations in cancer-related genes (Bettegowda et al., 2014), which is highly important for the early detection of cancers (Song et al., 2022).

### 2.2 Sample collection procedure

#### 2.2.1 Blood collection

As vital biological materials, blood samples are most frequently collected for cfDNA analysis due to the body’s reliance on blood circulation for metabolism. To achieve more reliable results, the choice between serum or plasma as the cfDNA analysis matrix is crucial (Kumar et al., 2018). Research suggests a preference for plasma in cfDNA analysis, as it helps circumvent the effects of genomic DNA (gDNA) released by leukocyte lysis on the concentration and purity of cfDNA (Martignano, 2019; Pittella-Silva et al., 2020). Although several studies reported higher cfDNA concentrations in serum than in plasma due to DNA degradation and contamination of gDNA from white blood cells (Wong et al., 2016; Li et al., 2017; Huang et al., 2020), cfDNA in plasma has proven to be more stable over time (Board et al., 2008). Moreover, a study aimed at evaluating the positive rate of epidermal growth factor receptor (EGFR) mutations in cfDNA revealed greater sensitivity when using plasma rather than serum (Vallee et al., 2013), underscoring the reliability of plasma-derived cfDNA.

Collection tubes with superior performance are preferred for blood collection. Anticoagulants, key components of blood collection tubes, can impact the quality and integrity of cfDNA (Luo et al., 2022). Widely used anticoagulants such as EDTA, citrate, and heparin exhibit different functional characteristics (Akat et al., 2019). Previously, EDTA tubes were commonly considered the standard for cfDNA analysis because EDTA inhibits DNase (Barra et al., 2015) and demonstrates better storage effects than heparin or citrate for delayed blood processing (Lam et al., 2004). Evidence suggests that plasma samples collected in EDTA tubes and processed within 6 h are most suitable for ctDNA analysis (Kang et al., 2016). However, when blood processing is delayed due to long-distance transportation or other unavoidable circumstances, these collection tubes may not preserve samples efficiently. This has led to the development of specialized blood collection tubes designed to preserve samples for extended periods (Sorber et al., 2020).

Recently, various specialized collection tubes with different properties have been widely utilized for blood collection for cfDNA analysis (Alidousty et al., 2017; Ungerer et al., 2020). While these specialized collection tubes (Table 2) vary slightly in specifications (e.g., volume and shape), they extend the storage time of blood samples without the need for controlled environmental conditions (Rothwell et al., 2016; Schmidt et al., 2017; Parackal et al., 2019; Ward Gahlawat et al., 2019; Zhao et al., 2019; Salvianti et al., 2020). To ascertain the tubes’ ability to maintain cfDNA levels during transportation or temporary storage, studies have compared them with each other or with EDTA tubes. Overall, the specialized tubes outperform EDTA tubes in preventing gDNA contamination and extending storage time (van Ginkel et al., 2017; de Kock et al., 2019). Furthermore, these specialized tubes maintain the quality of cfDNA samples within 3 days equally well, but their storage effects reportedly differ slightly after more than 7 days (Zhao et al., 2019).

**TABLE 2:** Recommended specialized blood collection tubes and storage conditions for cfDNA analysis.

Brands	Country	Volume (mL)	Temperature	Maximum storage time (day)	Explanations	References
Roche	Germany	8.5	20°C–30°C	7	More capable for preventing cfDNA contamination caused by white blood cells	Zhao et al. (2019)
Streck	United States of America	10	RT	14	Maintains the stability for up to 14 days post collection	Parackal et al. (2019)
PAXgene	Germany	10	15°C–25°C	7	Suitable for PCR-based quantification of total amount of cfDNA and for methylation analysis	Schmidt et al. (2017)
ImproGene	China	10	4°C–30°C	7–14	More sensitive in the detection of low frequency mutations	Salvianti et al. (2020)
Norgen	Canada	8.4	RT	7	Superior for cfDNA yield	Ward Gahlawat et al. (2019)
CellSave	United States	10	RT	4	Enables the analysis of both cfDNA and CTCs from the same tube	Rothwell et al. (2016)

RT, room temperature.

Some often overlooked variables during blood sample collection can impact cfDNA analysis. Proper needle selection is necessary, as excessively thin needles can cause hemolysis (Mouser et al., 2017). When collecting blood samples from children, professional collectors can enhance the efficiency of blood collection and minimize discomfort to the participants (Simundic et al., 2018). To prevent hemolysis, the tourniquet should be correctly positioned and not left in place too long during blood drawing (Phelan et al., 2018). The recommended duration for tourniquet application is generally within 1 min (Wall et al., 2014), as prolonged tourniquet use can lead to blood sample concentration and hemolysis (Jacob et al., 2021). Even though these operational details affecting cfDNA quality have not been fully investigated or described in the current literature, they should be considered during blood collection.

#### 2.2.2 Urine collection

Collecting urine samples is a completely noninvasive process typically carried out by the donors themselves. Urine collection is more convenient than blood collection, provided that there is good communication with donors beforehand (Itoh et al., 2013). Different types of urine samples, such as 24-h, morning, and random samples, are collected for various purposes (24-h and random urine for the urinary biochemical parameters, and morning urine for the extraction of tumor markers) (Cook et al., 2000; Shojaei-Far et al., 2017; Zhang et al., 2018). Morning urine is frequently preferred for cfDNA analysis due to its more stable total cfDNA content compared to that of other urine types (Zhou et al., 2021). This is because factors that might alter cfDNA content, such as the donor intentionally drinking excessive water before collection, are hard to control. Therefore, morning urine collection tends to be less affected.

The choice of suitable collection containers is also crucial, as they need to be user-friendly for donors. Ideally, these containers should be sterile (Sigdel et al., 2013; Cheng et al., 2017) and have a lid to prevent leakage. The donor’s gender, age, and physical condition should be considered when selecting an appropriate container. In addition, it is also recommended to immediately divide the collected urine into multiple portions to avoid freeze-thaw cycles that could affect the quality of the ucfDNA (Jordaens et al., 2023).

The question of whether to add protective agents to the collected urine samples is important. Taking cues from the practice of prolonging the storage time of blood samples collected in tubes with protective agents, adding additives to urine may enhance the stability of cfDNA (Murugesan et al., 2019). EDTA is most commonly used as a protective agent added to urine for optimal cfDNA storage outcomes (Lee et al., 2020). In a study comparing the extraction methods of short cfDNA fragments from urine, a 10 mmol/L EDTA solution was added to the collected urine to enhance analytical result accuracy (Oreskovic et al., 2019). Besides EDTA, the Streck reagent has also been used as a urine preservative for cfDNA protection (Murugesan et al., 2019). However, numerous studies have not clearly outlined whether protective agents were added to the collected urine (Table 4). Further research is necessary to enhance the effectiveness of preservatives in preserving ucfDNA. It is possible to find inspiration from the key components present in specialized collection tubes designed for the extended preservation of blood cfDNA. Moreover, the methods of long-term preservation of DNA might be beneficial to develop more effective preservatives for preserving ucfDNA.

### 2.3 Sample transportation before processing

Due to a lack of necessary equipment or well-trained professionals, samples often cannot be processed immediately after collection and have to be transported. Unpredictable conditions, such as violent shaking during transportation, prolonged transportation, or high temperature during temporary storage, may negatively impact sample quality and cfDNA analysis.

#### 2.3.1 Blood transportation

During transportation, stirring or violent vibration may cause blood sample hemolysis, resulting in the release of cell metabolites that inhibit the Taq enzyme activity and decrease PCR amplification efficiency (Ellervik and Vaught, 2015). As reviewed in El Messaoudi et al. (2013), cfDNA concentration slightly increased in blood samples stirred for 3 h at room temperature (El Messaoudi et al., 2013). Hence, significant or prolonged vibration should be avoided during blood sample transportation, especially at room temperature (the duration of stable cfDNA level is much longer in EDTA tubes when stored at 4°C than at room temperature) (Hidestrand et al., 2012).

In addition to blood collection tubes, the time and temperature of blood transportation postcollection should be controlled. A study comparing three collection tubes (K2EDTA, Roche, and Streck) revealed that Roche and Streck tubes were similarly effective in preventing gDNA release after 7 days of storage at room temperature, while K2EDTA tubes resulted in significant gDNA release (Zhao et al., 2019). Therefore, specialized tubes should be used when processing steps have to be delayed (Zhang et al., 2022). Hidestrand et al. reported that the samples should avoid being exposed to extreme temperatures during transportation by comparing the total cfDNA in plasma of EDTA samples and BCT samples at room temperature and 4°C (Hidestrand et al., 2012). As indicated in Table 3, the recommended transportation temperature is 4°C for EDTA tubes or room temperature for specialized tubes.

**TABLE 3:** Summary of used/recommended temporary storage conditions and different centrifugal procedures after collecting blood for cfDNA analysis.

Tube types	Temporary temperature after collection	Processing deadline	First centrifugation step	Second centrifugation step	Temporary temperature after centrifugation (°C)	Explanations	References
EDTA	ND	3 h	2,500 g, 10 min, RT	2,500 g, 10 min, RT	−80	The supernatant of 1–2 mL aliquots was stored at −80°C	Nuzzo et al. (2020)
EDTA	ND	2 h	380 g, 20 min	20,000 g, 10 min	−80	cfDNA is isolated from plasma by Hamilton Microlab STAR	van Dorp et al. (2023)
EDTA	RT	2 h	2000 g, 10 min, 4°C	16,000 g, 10 min, 4°C	−80	Roche is capable for preventing cfDNA contamination due to white blood cell disruption within 14 days	Zhao et al. (2019)
Roche	RT	7 d					
Streck	RT	7 d					
EDTA	AT	4 h	2000 g, 10 min, RT	2000 g, 10 min, RT	−80	No significance difference in the yields of cfDNA between the 4 h-EDTA, 4 h-CellSave and 96 h-CellSave samples	Rothwell et al. (2016)
CellSave	AT	4 h					
CellSave	AT	96 h					
EDTA	RT	24 h	380 g, 20 min, RT	20,000 g, 10 min, RT	−80	Lysis of white blood cells in blood samples increases with increasing centrifugation force	van Ginkel et al. (2017)
Streck	RT	5 d					
CellSave	RT	5 d					
EDTA	RT	4 h	820 g, 10 min, RT	16,100 g, 10 min, RT	−80	PAXgene tube is preferred in clinical practice for the scenario that samples are stored for over 24 h	de Kock et al. (2019)
PAXgene	RT	5 d	1900 g, 15 min, RT				
EDTA	AT	1 h	820 g, 10 min, RT	16,000 g, 10 min, RT	−80	No significant difference between collection protocols by measuring cfDNA yield and fragment size	Markus et al. (2018)
Streck	AT	24/72 h					
EDTA	AT	4 h	1,600 g, 10 min, 4°C	1,600 g, 10 min, 4°C	−80	Recommend to store blood samples less than 4 h at ambient temperature or 24 h at 4°C	Gerber et al. (2020)
EDTA	4°C	24 h					
EDTA	4°C	24 h	3,000 g, 10 min, RT	ND	−80	If plasma cannot be separated within 4 h or stored at 4°C, it should be collected in Streck before processing	Nesic et al. (2021)
Streck	RT	14 d	1,600 g, 10 min, RT	16,000 g, 10 min, RT			
EDTA	AT	24 h	1711 g, 10 min, RT	12,000 g, 10 min, RT	−80	cfDNA extracted from CellSave and EDTA can be used for methylated DNA sequencing	Deger et al. (2021)
CellSave	AT	96 h					

ND, no description; AT, ambient temperature; RT, room temperature.

#### 2.3.2 Urine transportation

The urine sample transportation temperature should be regulated based on different transportation distances. For distances that allow urine samples to be processed within 90 min, samples can be stored at room temperature for transportation (Eisinger et al., 2013; Dolscheid-Pommerich et al., 2016). For longer distances, measures such as using ice packs to maintain the urine samples at approximately 4°C or adding preservatives to urine samples (Ercan et al., 2015) should be implemented to prevent changes in physical and chemical properties of urine or the degradation of cfDNA fragments. The long-distance transportation may lead to the instability of cfDNA level in urine, which is equivalent to prolonging the temporary storage time before sample processing. Therefore, reliable transportation methods (e.g., equipping with enough ice packs or adding suitable preservatives) should be prioritized, especially when transferring rare disease samples. Additionally, it is necessary to prevent the rupture of the urine collection tube due to shock or external force during transportation, which might lead to urine sample leakage.

### 2.4 Temporary storage before sample processing

#### 2.4.1 Blood temporary storage

Research has shown that the storage temperature and delay time during temporary storage before sample processing significantly impact cfDNA concentration, fragments, and purity (Gerber et al., 2020). The concentration of cfDNA slightly increases with time during temporary storage (Bronkhorst et al., 2015; Bhangu et al., 2017). However, the unified delay time has not yet been determined.

Previous studies have investigated blood samples collected in EDTA tubes and processed within specific times, such as 1 h (Markus et al., 2018), 2 h (Zhao et al., 2019), 3 h (Nuzzo et al., 2020), 4 h (Gerber et al., 2020), 6 h (El Messaoudi et al., 2013) or 24 h (Nesic et al., 2021). However, a consensus on the maximum permissible delay time for processing has not been reached. Table 3 shows that the storage temperature of blood correlates with the delayed processing time, suggesting that blood samples should be processed within the delayed processing time corresponding to the specific storage temperature as soon as possible to ensure the optimal quality of cfDNA. Otherwise, it is recommended that blood samples collected in EDTA tubes should be stored at 4°C, and the delay time should not exceed 24 h (Van Paemel et al., 2021). The blood samples collected by specialized collection tubes are temporarily stored for a longer time than EDTA tubes, and the results are summarized in Tables 2, 3.

#### 2.4.2 Urine temporary storage

The delay time after urine collection is also different in previous studies, including 0 h (Janovicova et al., 2023), 1 h at 4°C (Cheng et al., 2017), 3 h (no description of temperature) (Nuzzo et al., 2020) and 24–72 h (no description of temperature) (Wever et al., 2022). The temporary storage conditions of urine samples after collection were usually neglected, so detailed descriptions of the variables were not provided in most related studies (Table 4). Due to the lack of comparative studies on the temporary storage conditions of urine samples, it is difficult to establish standard operational consensuses. The concentration of ucfDNA is greatly dependent on the addition of urine preservatives (Lee et al., 2020) during temporary storage. Table 4 shows that urine samples with EDTA can be temporarily stored for a longer period of time. Adding preservatives after urine collection is extremely important for sample stability (Pages et al., 2022), which makes the temporary storage conditions more variable and flexible.

**TABLE 4:** Summary of used/recommended temporary storage conditions and centrifugal procedures for urine cfDNA analysis.

Containers	Volume (mL)	Additives	Temporary temperature after collection	Processing deadline	First centrifugation step	Second centrifugation step	Temporary temperature after centrifugation	Explanations	References
Sterile cups	100	NA	ND	ND	200 g, 10 min	1800 g, 10 min	ND	DNA yields vary greatly between different individuals on different days	Streleckiene et al. (2018)
Sterile bottles	30–50	ND	4°C	1 h	3,000 g, 10 min, 4°C	ND	−80°C	Avoid collecting early morning urine	Cheng et al. (2017)
Sterile containers	50–100	ND	RT	1 h	2000 g, 20 min, RT	ND	−80°C	The pH of supernatant was adjusted to 7.0 using Tris-HCl before storage	Sigdel et al. (2013)
Sterile container	ND	NA	ND	3 h	2,500 g, 10 min, RT	ND	−80°C	ND	Nuzzo et al. (2020)
ND	ND	ND	ND	at once	1,600 g, 10 min, 4°C	16,000 g, 10 min, 4°C	ND	Midstream urine samples were processed right after urine collection	Janovicova et al. (2023)
Cell Preservation Solution Kit	10	ND	RT	72 h	1,000 g, 10 min	ND	ND	Urine was collected between the first morning urination and operative treatment	Zeng et al. (2020)
ND	ND	ND	−80°C	ND	2000 g, 10 min, 4°C	16,000 g, 10 min, 4°C	ND	Fresh urine was immediately stored at −80°C after collection	Ohta et al. (2021)
ND	ND	EDTA	ND	1 h	1,500 g, 10 min, 4°C	20,000 g, 10 min, 4°C	−80°C	ND	Mouliere et al. (2021)
Receptacles	20–30	EDTA	−20°C	ND	10,000 g, 15 min, 4°C	10,000 g, 15 min, 4°C	ND	Participants were instructed to collect the midstream urine	Zhu et al. (2021b)
Large container	300	NA	ND	24–72 h	3,000 g, 15 min	ND	−20°C	Ambulant urine collection was realized by a collection kit	Wever et al. (2022)
Collecting pipes	30	EDTA	ND	24–72 h	3,000 g, 15 min	ND	−20°C		

ND, no description; NA, no additive added to the urine; RT, room temperature.

### 2.5 Sample processing procedure

Ensuring that plasma and urine supernatant used for extracting cfDNA are free from cellular components, various specific protocols for processing blood and urine samples have been developed (Tables 3, 4). The main factors that may result in DNA contamination during sample processing include centrifugation speeds, centrifugal temperature, single or double centrifugation steps, and the duration of the centrifugation steps (Ungerer et al., 2020; Shin et al., 2022). These preanalytical variables can affect the effectiveness of blood and urine processing to varying degrees. However, no consensus has been reached regarding these preanalytical variables in the current sample processing protocols. Therefore, there is an urgent need to develop a unified and effective centrifugation protocol for processing blood and urine samples.

#### 2.5.1 Blood processing

Whole blood samples are primarily processed through centrifugation steps to remove cellular components, thus avoiding gDNA contamination of cfDNA (Martignano, 2019). The parameters involved in these steps, such as centrifugal force, temperature, number of centrifugations, and duration of centrifugation, can potentially introduce sample contamination. Therefore, it is crucial to determine the optimal parameters for a centrifugation scheme that can be universally applied to blood processing. Previous studies have indicated that the number of centrifugation steps and the centrifugal force are the key parameters in developing a centrifugation scheme for blood processing (de Kock et al., 2019; Zhao et al., 2019). These parameters should be given more attention than the temperature or duration of centrifugation steps to prevent sample contamination. Currently, a well-established approach for obtaining cell-free plasma fractions during blood processing involves an initial centrifugation step with lower centrifugal force followed by a subsequent second centrifugation step with higher centrifugal force. Moreover, the yield of plasma cfDNA did not differ after the third centrifugation step at 16,000 g compared to the second centrifugation step. Therefore, double centrifugation is widely recognized and used in current blood sample processing protocols to achieve satisfactory cfDNA analysis results (Volckmar et al., 2018; Sorber et al., 2019).

Centrifugal parameters for blood samples vary significantly and are summarized in Table 3. In the first centrifugation step, a slow centrifugal force, primarily ranging from 380–3,000×g for 10 min, is used to remove a large number of cell components (Nesic et al., 2021; van Dorp et al., 2023). In the second centrifugation step, a faster centrifugal force, mainly ranging from 12,000–20,000×g for 10 min, is usually performed to remove cellular residues and debris (Deger et al., 2021; van Dorp et al., 2023). These centrifugation steps are generally carried out at 4°C (Zhao et al., 2019; Gerber et al., 2020) or room temperature (Markus et al., 2018; de Kock et al., 2019).

#### 2.5.2 Urine processing

ucfDNA degrades more easily than blood-derived cfDNA due to urinary nucleases and contaminants (Yao et al., 2016); therefore, collected urine samples should be processed as soon as possible. Similar to blood samples, collected urine typically undergoes single or double centrifugation to remove cellular components or cell debris (Casadio and Salvi, 2019). However, the range of centrifugal force for urine samples is much larger than that for blood samples, as summarized in Table 4. Single centrifugation usually takes 10–20 min at speeds ranging from 1,000–3,000×g (Cheng et al., 2017; Zeng et al., 2020). The double centrifugation procedure consists of a first centrifugation step at 200–2000×g for 10 min, followed by a faster second centrifugation step at 1800–16,000×g for 10 min (Streleckiene et al., 2018; Mouliere et al., 2021; Ohta et al., 2021). These centrifugation steps are also carried out at 4°C (Cheng et al., 2017; Zhu et al., 2021) or room temperature (Sigdel et al., 2013; Nuzzo et al., 2020).

In some studies, urine samples were directly frozen at −20°C or −80°C after collection without a centrifugation step (Kim et al., 2022; Janovicova et al., 2023). The frozen samples must be thawed for subsequent processing or analysis, which can result in cell lysis in urine during the freeze-thaw cycle (Luo et al., 2018). In a study by Oreskovic A et al. on the diagnostic accuracy of a tuberculosis cfDNA test using sequence-specific purification of ucfDNA, the collected urine samples underwent several stages, including freezing at −20°C at the collection point, transportation on dry ice, freezing at −80°C, and thawing at 37°C before centrifugation (Oreskovic et al., 2021). However, the study did not describe or discuss whether the urine samples were immediately processed or not, nor the potential effect of the above steps on the urine. All preanalytical variables may affect the quality and final analysis of cfDNA, which should be clearly described in each study.

### 2.6 Temporary storage between sample processing and extraction

Many studies do not immediately proceed to cfDNA extraction after centrifuging the collected blood or urine samples. This delay is often due to specific experimental purposes or the need for centralized cfDNA extraction. The conditions of temporary storage, such as the duration and temperature between sample processing and extraction, are vital variables that could impact cfDNA quality. Cellular components and cell debris are removed from the samples during centrifugation, suggesting that changes in cfDNA likely result from DNA fragment degradation during temporary storage (Ellervik and Vaught, 2015). However, temporary storage conditions are not yet standardized.

After centrifugation, the majority of the collected blood and urine samples were frozen at −80°C until DNA extraction, as summarized in Tables 3, 4. One study showed that cfDNA concentration increased slightly when the centrifuged plasma samples were stored at room temperature for varying lengths of time, ranging from 0 to 4 h, before extraction (El Messaoudi et al., 2013). Another study revealed that cfDNA fragmented gradually over 3 months when centrifuged plasma was stored at −20°C (Bronkhorst et al., 2015). The plasma used for detecting specific DNA sequences can be stored at −80°C for up to 10 years, while samples for quantitative analysis can only be stored at −80°C for 9 months (Diao et al., 2022).

Specialized kits allow the collected urine to be temporarily stored for a longer period of time before extraction. Zeng et al. reported that the Cell Preservation Solution Kit was used to collect urine samples and allowed the samples to be transferred to the laboratory for processing within 72 h at room temperature (Zeng et al., 2020). In another more detailed report, urine samples were collected using the specialized kits including a large collection container (300 mL) and three 30 mL collection tubes and then transported to the Department of Pathology of Amsterdam UMC (Wever et al., 2022). Importantly, 2 mL of 0.6 M EDTA as a preservative agent in the collection tubes allowed the samples to be processed within 24–72 h. Except for urine samples collected by specialized kits stored at −20°C after a single centrifugation or samples without relevant storage descriptions, the other collected urine samples were frozen at −80°C after single or double centrifugation until DNA extraction (Table 4). Studies specifically related to the temporary storage of collected urine samples are scarce. Nonetheless, the conditions summarized above for plasma samples are also applicable to urine samples that have undergone single or double centrifugation during temporary storage.

### 2.7 cfDNA extraction procedure

Efficient cfDNA extraction is essential for ensuring the accuracy and reliability of downstream analytical results. However, the extracted cfDNA can often be too fragmented or too low in content, possibly leading to regrettable analytical data or failed application. Thus, finding a way to efficiently and cost-effectively separate cfDNA from samples has been a central issue for researchers.

A wide array of extraction methods, including traditional (liquid-phase-based or solid-phase-based DNA isolation methods) (Janku et al., 2021), improved (methods for separating cfDNA mainly based on chromatographic columns or magnetic beads) (Lin et al., 2021), and novel (methods for separating cfDNA using new technologies or materials) (Liu et al., 2022) technologies, as well as manual (Wang et al., 2021) or automatic (Lee et al., 2018) methods, have been employed to extract cfDNA. These methods vary in terms of recovery efficiency, fragment discrimination, and reproducibility (Ungerer et al., 2020), making it challenging to select the optimal method for cfDNA isolation. Factors such as the efficiency of extracting low-content DNA (Lee et al., 2018), purity (Uwiringiyeyezu et al., 2022), repeatability (Letendre and Goggs, 2017), and cost (Diefenbach et al., 2018) are usually considered when applying extraction protocols. Commercial specialized kits based on current optimized technologies seem to offer clear advantages for cfDNA extraction (Janovicova et al., 2020) and are routinely used in many studies.

Currently, innovative technologies based on magnetic particles (van der Leest et al., 2022) or spin columns (Diefenbach et al., 2018) are the most common methods in specialized commercial kits for cfDNA extraction. A comparative study of a series of commercial kits analyzing artificially added DNA fragments showed that the Qiagen QIAamp circulating nucleic acid kit, based on a spin column, was the most stable kit (Diefenbach et al., 2018). However, the Qiagen QIAamp kits have a significant shortcoming: some short DNA fragments are lost during extraction and purification, resulting in a decrease in cfDNA yield (Kemp et al., 2014). Comparatively, the kits based on magnetic particles for cfDNA isolation have a higher recovery rate for short cfDNA fragments (50–250 bp) than those based on silica membranes (Markus et al., 2018; Ungerer et al., 2020).

#### 2.7.1 Kits for extracting blood-derived cfDNA

Several manufacturers, such as Qiagen, Norgen, Thermo, and Promega, produce specialized commercial kits for extracting blood-derived cfDNA (Diefenbach et al., 2018; van Dessel et al., 2019; Huebner et al., 2021; Wang et al., 2021). Among these, Qiagen’s systematic kits are the most commonly used (Vermeulen et al., 2017; Jain et al., 2019; Jiang et al., 2020; Kallionpaa et al., 2021), However, comparing the performance of these kits is challenging due to variations in sample collection, processing, and analysis procedures (Table 5). For example, when PCR is used to quantify specific genes, the sensitivity may decrease or even vanish as cfDNA fragments become shorter (Ungerer et al., 2020). Nonetheless, a few of the few studies have compared these kits under identical conditions. Devonshire et al. compared the extraction efficiency of four commercial kits (QIAamp circulating nucleic acid kit, NucleoSpin Plasma XS kit, FitAmp plasma/serum DNA isolation kit, and QIAamp DNA blood mini kit) using quantitative PCR measurements of seven different reference genes (Devonshire et al., 2014). They found that the extraction efficiency of the kits was in the following order: QIAamp circulating nucleic acid kit > QIAamp DNA blood mini kit > NucleoSpin Plasma XS kit > FitAmp plasma/serum DNA isolation kit. The QIAamp circulating nucleic acid kit and NucleoSpin Plasma XS kit were more efficient in extracting short DNA fragments than the QIAamp DNA blood mini kit.

**TABLE 5:** Summary of kits for blood-derived cfDNA extraction and storage conditions of extracted cfDNA.

Product	Manufacturer	Sample volume	Storage conditions	Quantitative methods	Finding	References
QIAmp Circulating Nucleic Acid Kit	Qiagen	3–8 mL	−80°C	dPCR using the KRAS G12/G13 Screening Multiplex Kit	The cfDNA-extraction conditions lead to higher cfDNA concentrations	de Kock et al. (2019)
Qiagen Circulating Nucleic Acids Kit	Qiagen	1 mL	−80°C	Qubit dsDNA High Sensitivity Assay Kit	ND	Nuzzo et al. (2020)
Maxwell RSC ctDNA Plasma Kit	Promega	1 mL	ND	Qubit and ddPCR	ND	Ohta et al. (2021)
QIAamp Circulating Nucleic Acid Kit	Qiagen	ND	ND	Qubit	Plasma stored at −80°C is suitable for NGS	Jiang et al. (2020)
QIAamp Circulating Nucleic Acid Kit	Qiagen	2 mL	ND	Qubit HS Assay Kit and Qubit	ND	Kallionpaa et al. (2021)
Maxwell RSC ccfDNA plasma kit	Promega	ND	ND	Fluorometric measurement and qRT‒PCR of ALU and mtDNA fragments	A higher ccfDNA yield by Maxwell kit and a higher ccfDNA integrity by QIAamp kit	Huebner et al. (2021)
QiAamp minElute ccfDNA mini kit	Qiagen	ND	ND			
QIAamp DSP Virus Kit	Qiagen	1 mL	ND	dPCR	The QIAamp Circulating Nucleic Acid Kit delivered a significantly higher yield	Jain et al. (2019a)
QIAamp Circulating Nucleic Acid Kit	Qiagen	1 mL	ND			
QIAamp Circulating Nucleic Acid Kit	Qiagen	2 mL	ND	Quantitative methylation specific PCR (qMSP) and dPCR (KRAS, TP53, and PIK3CA mutations)	Both QIAamp Kit and Maxwell^®^ RSC Kit were compatible with MeD-seq analysis, whereas the QiaSymphony DSP Kit yielded considerably fewer reads compared to the QIAamp kit	Deger et al. (2021)
Maxwell^®^ RSC ccfDNA Plasma Kit	Promega	2 mL	ND			
QiaSymphony DSP Circulating DNA Kit	Qiagen	2 mL	ND			
QIAamp Circulating Nucleic Acid Kit (QA)	Qiagen	2 mL	ND	Qubit and qPCR (TERT)	The QS automated platform has comparable performance to the QA and outperformed the MX platform	van Dessel et al. (2019)
QIAsymphony SP Circulating DNA Kit (QS)	Qiagen	2 mL	ND			
Maxwell ccfDNA Plasma Custom Kit (MX)	Promega	2 mL	ND			
QIAamp Circulating Nucleic Acids kit (QA)	Qiagen	1 mL	−70°C	qPCR (Alu sequences)	QA and DSP kit both efficiently purify DNA regardless of fragment size, whereas QD kit only effectively extract high molecular weight DNA. QU Kit produced the lowest yields	Warton et al. (2018)
QIAamp DNA Blood Mini kit (QD)	Qiagen	1 mL	−70°C			
QIAamp Ultrasens Virus kit (QU)	Qiagen	1 mL	−70°C			
QIASymphony DSP Virus kit (DSP)	Qiagen	1 mL	−70°C			
QIAamp circulating nucleic acid kit	Qiagen	4 mL	ND	Qubit, qPCR (EGFR gene mutations) and Agilent 2,100 Bioanalyzer	QIAamp circulating nucleic acid kit and Microdiag^®^ circulating DNA kit had the highest recovery rate for short DNA fragments	Wang et al. (2021)
AmoyDx^®^ Circulating DNA kits	Amoy Diagnostics	4 mL	ND			
Microdiag^®^ circulating DNA isolation kit	MicroDiag	2 mL	ND			
MagMAX cell-free DNA isolation kit	Thermo	2 mL	ND			
QIAamp circulating nucleic acid kit	Qiagen	1–5 mL	ND	ddPCR	The Qiagen QIAamp circulating nucleic acid kit was the most consistent performing kit. The Qiagen QIAamp minElute ccfDNA mini kit displayed the best performing magnetic bead-based kit with a simpler workflow	Diefenbach et al. (2018)
Plasma/serum cell-free circulating DNA Purification midi kit	Norgen Biotek	1–4 mL	ND			
QIAamp minElute ccfDNA mini kit	Qiagen	1–2 mL	ND			
Maxwell RSC ccfDNA plasma kit	Promega	1 mL	ND			
MagMAX cell-free DNA isolation kit	Applied Biosystems	0.1–10 mL	ND			
NextPrep-Mag cfDNA isolation kit	Bioo Scientific	1–3 mL	ND			

ND, no description.

Warton K et al. evaluated four commercial DNA purification kits (QIAamp Circulating Nucleic Acids kit, QIAamp Ultrasens Virus kit, QIAamp DNA Blood Mini kit, and QIASymphony DSP Virus kit) for the extraction of low- (115 base) and high-molecular-weight DNA (247 base) from plasma by qPCR quantification of endogenous Alu sequences (Warton et al., 2018). The study revealed that both the Circulating Nucleic Acids kit and the QIASymphony DSP Virus kit efficiently extracted DNA from plasma regardless of the size of DNA fragments, while the DNA Blood Mini kit only effectively extracted high-molecular-weight DNA. Overall, the QIAamp Circulating Nucleic Acids kit is the most widely used product with better performance than other kits for cfDNA analysis.

In summary, although the Qiagen Company kits lost short DNA fragments during the extraction procedure, they showed relatively higher efficiency and recovery rates (Wang et al., 2021). Additionally, long duration of storage before sample processing may cause cell lysis, resulting in a higher total amount of cfDNA. Therefore, the selection of kits should be based on the specific analytical targets (Table 5).

#### 2.7.2 Kits for extracting urine-derived cfDNA

The extraction protocols of cfDNA in many studies were originally developed mainly for extracting high-integrity gDNA from blood or virus particles rather than highly fragmented cfDNA (Repiska et al., 2013). Therefore, how to efficiently extract cfDNA from urine is usually neglected. Apart from cfDNA originating from exfoliated urothelial cells, the peak length of urine-derived cfDNA depends on glomerular filtration, which requires further degradation of all cfDNA fragments before entering the urine (Yao et al., 2016). While the peak length of plasma cfDNA is 160–167 bp, most urine cfDNA fragments are less than 100 bp (Burnham et al., 2018). Therefore, kits designed for blood-derived cfDNA extraction may not be suitable for urine-derived cfDNA isolation (Oreskovic et al., 2019). Currently, specialized commercial kits for extracting urine-derived cfDNA are offered by manufacturers such as Qiagen, Norgen, Thermo, Promega, and PerkinElmer (Table 6). Lee EY et al. compared the efficiency of four commercial kits (Urine Cell-Free Circulating DNA Purification Midi Kit, Quick-DNA™ Urine Kit, QIAamp Circulating Nucleic Acid Kit, and MagMAX™ Cell-Free DNA Isolation Kit) by an Agilent 2,100 Bioanalyzer for ucfDNA isolation and found that the QIAamp Circulating Nucleic Acid Kit and the MagMAX™ Cell-Free DNA Isolation Kit had the highest cfDNA yield within the 50–300 bp fragment range, while the MagMAX™ Cell-Free DNA Isolation Kit and the Urine Cell-Free Circulating DNA Purification Midi Kit had the highest cfDNA yield within the 50–100 bp fragment range (Lee et al., 2020). Another study by Oreskovic A et al. compared three commercial kits (Norgen, QIAamp, and MagMAX) for extracting short cfDNA fragments from urine (Oreskovic et al., 2019). The study showed that the Norgen kit exhibited a high recovery rate for short cfDNA fragments, although the kit resulted in PCR inhibition, while the other two kits had the lowest recovery rate for short cfDNA fragments. In summary, each kit for urine-derived cfDNA extraction has its own characteristics and advantages, emphasizing the importance of selecting a suitable cfDNA isolation kit based on the specific research purpose. In addition, for ucfDNA extraction, kits allowing the extraction of cfDNA from large-volume urine samples (usually greater than or equal to 10–15 mL) are recommended, as this facilitates obtaining a sufficient amount of ucfDNA (Casadio and Salvi, 2019; Martignano, 2019; Oreskovic et al., 2019; Janovicova et al., 2020).

**TABLE 6:** Summary of kits for urine-derived cfDNA extraction and storage conditions of extracted cfDNA.

Product	Manufacturer	Sample volume (mL)	Storage conditions	Quantitative methods	Finding	References
Circulating Nucleic Acid kit	Qiagen	3	ND	NanoDrop and Qubit	ND	Zhou et al. (2021)
Qiagen genomic DNA extraction Kit	Qiagen	2	−80°C	Qubit dsDNA HS Assay Kit	ND	Nuzzo et al. (2020)
Maxwell RSC circulating DNA Purification Kit	Promega	20	ND	Qubit and ddPCR	ND	Ohta et al. (2021)
NEXTprep-Mag Urine cfDNA Isolation Kit	PerkinElmer	4	ND	Agilent 2,200 and TapeStation Analysis Software	The bead-based method was tended to yield more cfDNA per ml of urine and PerkinElmer kit is more efficient at capturing short DNA	Streleckiene et al. (2018)
Urine Cell-Free Circulating DNA Purification Midi Kit	Norgen Biotek	10	ND			
JBS cfDNA extraction kit (kit J)	JBS Science	3	no storage or −20°C	JBS Artificial Spike-In DNA Quantification kit, TapeStation 4,200 system and qPCR	Kit J recovered remarkably more spike-in DNA than kit M or kit Q	Lin et al. (2021)
MagMAX Cell-Free DNA Extraction kit (kit M)	Thermo	3				
QIAamp Circulating Nucleic Acid Kit (kit Q)	Qiagen	3				
Norgen Urine Cell-Free Circulating DNA Purification Mini Kit	Norgen	2	ND	qPCR (DNA fragment length 40-, 80-, and 150-nt targets)	The Norgen kit resulted in consistent PCR inhibition but had high recovery of short fragments. The QIAamp and MagMAX kits had minimal recovery of fragments <150 and <80 nt, respectively. The methods vary widely in ability to capture short fragments	Oreskovic et al. (2019)
Qiagen QIAamp Circulating Nucleic Acid Kit	Qiagen	4	ND			
Thermo Fisher Scientific MagMAX Cell-Free DNA Isolation Kit	Thermo	1	ND			
QIAamp Circulating Nucleic Acid Kit (QC)	Qiagen	4	ND	DNA Chip-Based Agilent 2,100 Bioanalyzer	The NU kit was efficient for extraction of short fragments (50–100 bp) with the lowest genomic DNA contamination. Kit ZQ had the best cost-efficiency for obtaining the same amount of ucfDNA	Lee et al. (2020)
MagMAX™ Cell-Free DNA Isolation Kit (MM)	Thermo	4	ND			
Urine Cell-Free Circulating DNA Purification Midi Kit (NU)	Norgen Biotek	10	ND			
Quick-DNA™ Urine Kit (ZQ)	Zymo Research	24	ND			

ND, no description.

### 2.8 Quality control of extracted cfDNA

Quality evaluation of extracted cfDNA prior to analysis is critical. This involves assessing concentration, fragment size, and DNA Integrity Number (DIN). The quality control results may indicate the reliability of preanalytical procedures. Unexpected results often suggest potential issues with these procedures. Therefore, sensitive and accurate methods are required for cfDNA quantification.

qPCR-based techniques are also commonly used to quantify cfDNA.With their high sensitivity (Pan et al., 2017), accuracy (Leung et al., 2021), and low false positive rate (Yin et al., 2022), these methods can be used to measure trace nucleic acids effectively (Pan et al., 2017) and analyze cfDNA for known mutations (Hatipoglu et al., 2022). By detecting housekeeping genes (Aucamp et al., 2016) or noncoding repetitive sequences (Hussein et al., 2019) in cfDNA and fitting the standard curve with a reference substance (Tang et al., 2020), absolute cfDNA concentrations can be quantified using PCR-based methods. Frequently used reference genes include TERT (Akuta et al., 2020), GAPDH (Salinas-Sanchez et al., 2021), EGFR (Sugimoto et al., 2023), KRAS (Berchuck et al., 2022), and ALU (Shi et al., 2020). However, the lack of unified reference genes results in significant variations in the quantitative results of PCR-based methods, hindering efficient comparisons across different studies (Devonshire et al., 2014). Moreover, these methods are easily interfered with by compounds. Yokota et al. reported that heparin in plasma could inhibit Taq reaction in PCR analysis (Yokota et al., 1999). The use of cfDNA extracted from jaundice plasma occasionally interfered with PCR reaction, suggesting that a compound in jaundice plasma is not conducive to PCR analysis (Meddeb et al., 2019).

NGS, which greatly reduces sequencing costs and improves accuracy, can analyze millions of ctDNA molecules simultaneously and has been applied to ctDNA detection (Chen and Zhao, 2019). Although PCR-based methods are sensitive, inexpensive and do not require complex information, they are only able to detect known sequences (Taly et al., 2017). In contrast, NGS is high-throughput and can identify new genetic information, but the method is time-consuming and depends on complex data analysis (Postel et al., 2018).

Considering a wide range of applications and advanced technologies, Qubit and Bioanalyzer are currently the optimal methods for cfDNA quantification (Kumar et al., 2018). The Qubit fluorometer offers excellent analytical sensitivity (Burnham et al., 2018) and can simultaneously detect up to eight samples (Parackal et al., 2019). Compared to NanoDrop and qPCR-based methods, the Qubit fluorometer is a suitable compromise considering measurement precision, processing time, and cost simultaneously (Burnham et al., 2018; Khetan et al., 2019). The Bioanalyzer system from Agilent Technology provides detailed information on cfDNA fragment size and level (Lapin et al., 2018), and automatically provides DIN values ranging from 1 (highly degraded) to 10 (extremely intact) to quantitatively evaluate DNA integrity (Truszewska et al., 2020). This system is ideal for quality control of cfDNA samples utilized in NGS (Yu et al., 2021) and qPCR workflows (Hussing et al., 2018). The Cell-free DNA ScreenTape assay can be used to analyze cfNDA samples from 50 bp to 700 bp and detect high molecular weight DNA contaminations (Terp et al., 2024). However, Femtopulse, another Agilent Technology, is a powerful and effective pulsed field capillary electrophoresis system with high sensitivity (Hashem et al., 2020). The system can run for up to 88 samples for cfDNA analysis on a gel simultaneously and provides results in as little as 1.5 h, which is quicker and more economical than the use of a bioanalyzer.

A new technique that can detect cfDNA directly in plasma without prior DNA extraction was developed in 2018. The developers used this technology to analyze cfDNA and found that the measured cfDNA concentrations correlated with those measured by digital PCR (Andriamanampisoa et al., 2018). Further study demonstrated that the analytical performance of the technology is equivalent to that obtained after purification and concentration, with a precision of ∼1% for size features (Boutonnet et al., 2023). In addition, several emerging technologies, including sophisticated cellular biosensors (Cooper et al., 2023), electrochemical biosensors (Wang et al., 2022) and fluorescence-enhancing all-dielectric metasurface biosensors (Iwanaga et al., 2023), can detect the content of cfDNA without complicated processing. These methods with high sensitivity have advantages in low-content detection, but they are not suitable for more detailed analysis of DNA fragments, so they are not generally used for quality control of cfDNA analysis.

Overall, each method has advantages and disadvantages. PCR can accurately and sensitively detect trace amounts of DNA, but it is susceptible to interference from certain compounds. Bioanalyzer and Femtopulse are often used to analyze the fragments and concentration of cfDNA. A bioanalyzer cannot detect larger fragments that can be analyzed by qPCR, while some samples that cannot be amplified by qPCR can be detected by a bioanalyzer (Krasic et al., 2021). In summary, the comparison of cfDNA yields obtained by different quantitative methods is infeasible, and non-PCR methods can compensate for the deficiency of PCR methods (Akbariqomi et al., 2019).

### 2.9 Long-term storage of extracted cfDNA

If not immediately analyzed, extracted cfDNA should be stored at low temperatures. Long-term preservation is crucial to ensure effective downstream applications. Shorter cfDNA fragments may yield poor quantitative results (Cook et al., 2018), so factors causing DNA degradation or breakage during storage should be mitigated.

Chemical degradation poses the main threat to DNA preservation, so nuclease contamination should be avoided during sample processing and extraction (Ellervik and Vaught, 2015). Factors impacting cfDNA quality during long-term storage include storage temperature and duration, repeated freeze-thaw cycles, and storage tubes (Ungerer et al., 2020).

Long-term storage of cfDNA is typically performed at −20°C or −80°C (Nuzzo et al., 2020; Lin et al., 2021). The appropriate temperature depends on the requirements of subsequent applications. As reviewed in El Messaoudi et al. (2013), cfDNA should be stored at −20°C for less than 3 months for quantification and fragmentation analyses, while it can be stored at −20°C or −80°C for up to 9 months for mutation analyses (El Messaoudi et al., 2013). Low concentrations of cfDNA are more prone to degradation (Martignano, 2019), reducing the storage time.

Polypropylene tubes are recommended for storing cfDNA before freezing as they absorb less DNA (Meddeb et al., 2019). The walls of LoBind tubes may absorb cfDNA, leading to lower sample concentrations (Ungerer et al., 2020). After freezing, extracted cfDNA should not undergo more than three freeze-thaw cycles. Shao et al. (Shao et al., 2012) found that increasing freeze-thaw cycles accelerates DNA degradation, with larger DNA fragments degrading most readily. Increasing DNA concentration can reduce degradation caused by repeated freeze-thaw cycles.

## 3 Conclusion and future directions

Achieving consensus on the various variables in preanalytical procedures is critical for ensuring the reliability and repeatability of cfDNA measurements. Notable progress, including the development of analytical methods and specialized products, has been made in recent years. However, many variables associated with preanalytical procedures remain undefined or inconsistent or interact with each other, particularly in the case of urine samples. The guidelines for preanalytical variables of blood samples have been developed and summarized in recent years (Meddeb et al., 2019; Greytak et al., 2020; Lampignano et al., 2020), offering valuable insights and inspiration for the standardization of preanalytical procedures for urine-derived cfDNA analysis. For instance, the addition of EDTA to collected samples (Markus et al., 2021) and timely processing of samples (Zhang et al., 2022) is beneficial to enhance cfDNA quality, and these protocols are also applicable to urine-derived cfDNA.

**FIGURE 2:**
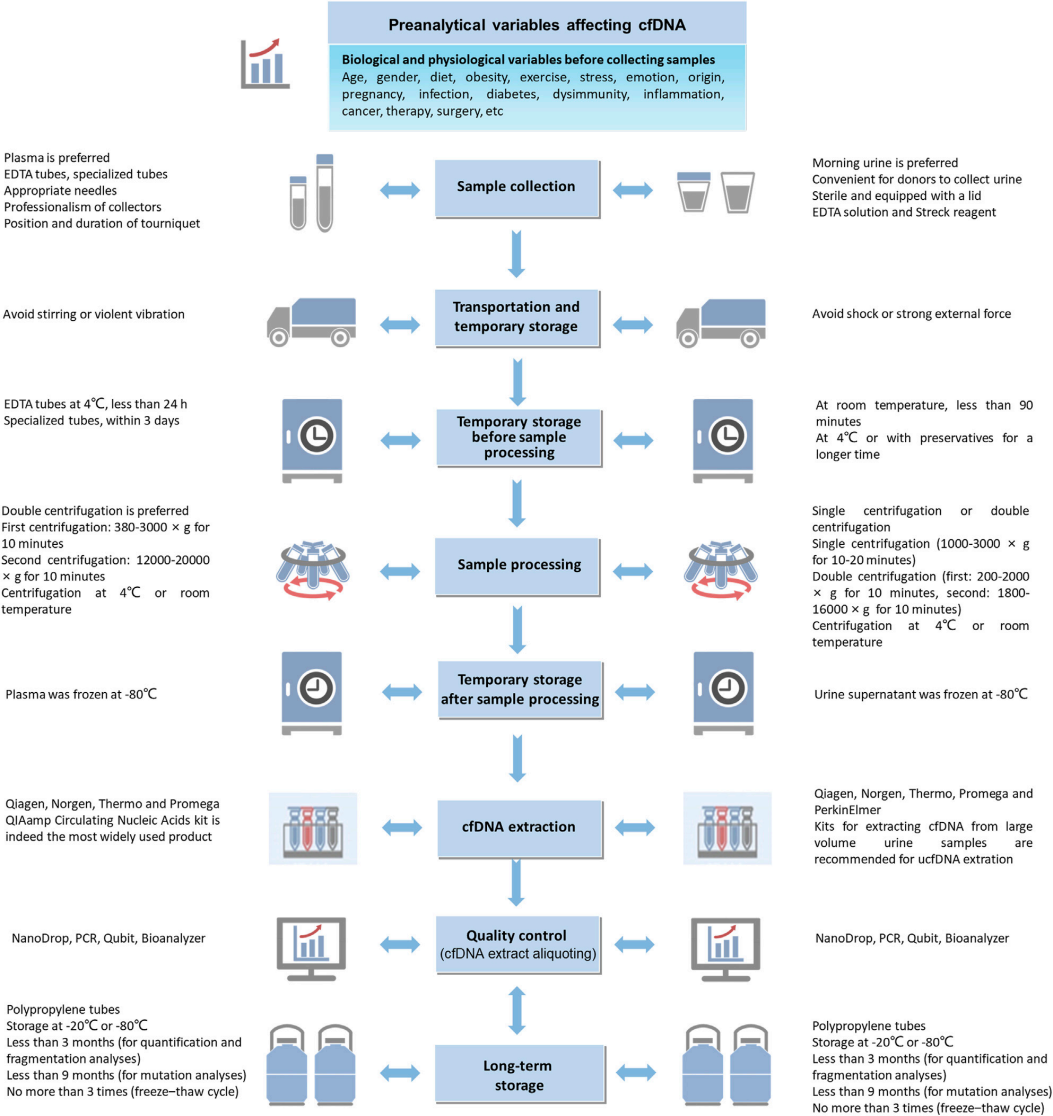
Preanalytical factors affecting cell-free DNA analysis from blood and urine samples.

This review discusses and summarizes the crucial variables in each preanalytical stage for analyzing blood-derived and urine-derived cfDNA (Figure 2). However, it is undeniable that achieving perfect coordination in the implementation of standardized preanalytical procedures is challenging due to objective conditions such as potential differences in funding and equipment resources among institutions or laboratories. Nevertheless, the following points can be explored further: (1) further verification of the impact of preanalytical variables on ucfDNA analysis; (2) development of multifunctional kits that efficiently extract short DNA fragments while preventing gDNA contamination; and (3) comparison and verification of measurement results from different internal reference genes when using PCR-based technologies for cfDNA quantification. These future directions will not only help address existing gaps in cfDNA preanalytical procedure standardization but also facilitate the broader and more accurate application of cfDNA analysis in clinical diagnostics and research.
